# A Novel Approach for Transcription Factor Analysis Using SELEX with High-Throughput Sequencing (TFAST)

**DOI:** 10.1371/journal.pone.0042761

**Published:** 2012-08-03

**Authors:** Daniel J. Reiss, Frederick M. Howard, Harry L. T. Mobley

**Affiliations:** 1 Department of Microbiology and Immunology, University of Michigan, Ann Arbor, Michigan, United States of America; 2 University of Michigan Medical School, Ann Arbor, Michigan, United States of America; Aligarh Muslim University, India

## Abstract

**Background:**

In previous work, we designed a modified aptamer-free SELEX-seq protocol (afSELEX-seq) for the discovery of transcription factor binding sites. Here, we present original software, TFAST, designed to analyze afSELEX-seq data, validated against our previously generated afSELEX-seq dataset and a model dataset. TFAST is designed with a simple graphical interface (Java) so that it can be installed and executed without extensive expertise in bioinformatics. TFAST completes analysis within minutes on most personal computers.

**Methodology:**

Once afSELEX-seq data are aligned to a target genome, TFAST identifies peaks and, uniquely, compares peak characteristics between cycles. TFAST generates a hierarchical report of graded peaks, their associated genomic sequences, binding site length predictions, and dummy sequences.

**Principal Findings:**

Including additional cycles of afSELEX-seq improved TFAST's ability to selectively identify peaks, leading to 7,274, 4,255, and 2,628 peaks identified in two-, three-, and four-cycle afSELEX-seq. Inter-round analysis by TFAST identified 457 peaks as the strongest candidates for true binding sites. Separating peaks by TFAST into classes of worst, second-best and best candidate peaks revealed a trend of increasing significance (e-values 4.5×10^12^, 2.9×10^−46^, and 1.2×10^−73^) and informational content (11.0, 11.9, and 12.5 bits over 15 bp) of discovered motifs within each respective class. TFAST also predicted a binding site length (28 bp) consistent with non-computational experimentally derived results for the transcription factor PapX (22 to 29 bp).

**Conclusions/Significance:**

TFAST offers a novel and intuitive approach for determining DNA binding sites of proteins subjected to afSELEX-seq. Here, we demonstrate that TFAST, using afSELEX-seq data, rapidly and accurately predicted sequence length and motif for a putative transcription factor's binding site.

## Introduction

**Figure 1:**
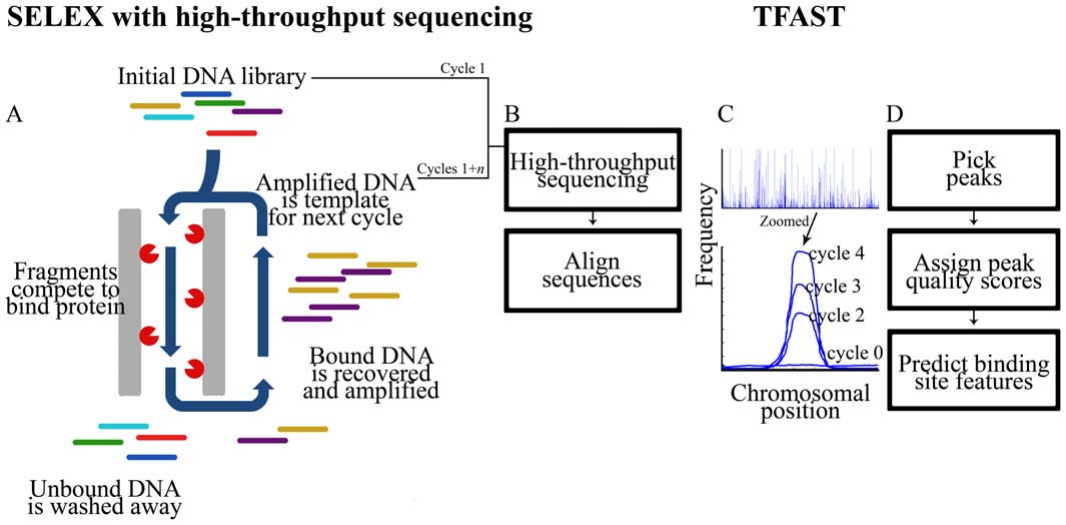
Schematic of the TFAST workflow. TFAST analyzes data produced using SELEX and high-throughput sequencing. (**A**) An overview of SELEX. Members of an input DNA fragment library compete to bind a protein of interest. Out-competed fragments are washed away and removed. Fragments that bind competitively are recovered and separated from the protein (*e.g.*, by phenol-chloroform extraction). Recovered fragments are amplified using low-cycle PCR, and the resultant library becomes the input for the next cycle. *n* cycles are repeated to enrich for strongly binding fragments. (**B**) DNA inputs for each cycle are subjected to high-throughput sequencing. Sequence reads are aligned to the relevant target genome, producing (**C**) frequency-position plots for each input sequenced. Shown is a magnified example of a region of the chromosome that behaves as a true binding site, enriching in frequency with each cycle. “Cycle 1” refers to the initial DNA library. (**D**) TFAST identifies and evaluates peaks and compares peak characteristics across all sequenced inputs. TFAST assigns quality scores to peaks and predicts binding site features. TFAST also generates quality scores on randomly selected regions of the chromosome to act as background controls, to improve downstream motif analysis.

Systematic Evolution of Ligands by Exponential Enrichment (SELEX) is a technique for determining nucleotide binding sites of transcription factors ([1], [2] and reviewed in [3]). SELEX is an iterative method where the products of one cycle are used to generate the input for the next (**Fig. 1a**), enriching strongly binding sequences in the output. In the past, only the terminal cycle was analyzed, and typically 50 or fewer members of the cloned output library were sequenced [1], [2]. High-throughput sequencing has made SELEX-seq possible, in which intermediate cycles are analyzed and millions of members of each cycle are sequenced. SELEX-seq improves the detection and analysis of binding sites over SELEX alone because behavior of the library can be scrutinized in million-fold greater detail, and between all cycles instead of only within a terminal cycle. SELEX-seq can detect the presence of library members and quantify their representation, making it possible to estimate binding affinities of many sequences and to compare sequences to one another to generate motifs [4], [5], [6], [7]. This advance also obviates the need to run the protocol until only a single sequence predominates in the terminal cycle. In fact, successful SELEX-seq experiments depend on multiple species being detectable in the terminal sequenced cycle to accurately infer binding characteristics [4].

Usually, the library of DNA to be enriched consists of random-sequence 10–20 bp oligonucleotides, called aptamers. This type of library is especially useful when a specific subset of aptamers is itself the desired end-product. Inter-cycle computational comparators (see [4], [5], [8] for examples) utilize the iterative nature of SELEX-seq to calculate binding affinity and activity of individual aptamers or aptamer sets, which is useful in designing therapeutic aptamers [9], [10], [11]. However, use of aptameric SELEX-seq for discovery of genomic binding sites may be problematic. Aptameric libraries are by definition random, so a given sequence may not be found within a target genome and thus discovered sequences may lack physiological relevance. Aptamers also lack genomic context, so that binding behavior may obscure *in vivo* findings by too strongly reflecting *in vitro* conditions [12].

To address these potential pitfalls of SELEX-seq, we designed an aptamer-free SELEX-seq protocol (afSELEX-seq) that uses sheared genomic dsDNA as the input library, with which we successfully identified a novel and unique transcription factor binding site [6]. Our approach incorporates the advantage of multiple-round enrichment with a physiologically relevant target library. In afSELEX-seq, the results of every sequenced cycle are aligned to a target genome, and alignments are compared between rounds to predict binding sites. Recently, there has been an exponential increase in the need for new software to meet the challenges of high-throughput sequencing output. As sequencing technologies have evolved and the size of datasets has increased, it has become necessary to automate data analysis, as by-hand approaches are becoming rate-limiting [13]. Performing a literature search through the NCBI for publications related to new software and sequence analysis reveals that such articles have nearly doubled every year in the last three years, highlighting the increased demand and opportunities within the scientific community. Unsurprisingly then, to process our results it was necessary to develop novel software capable of both analyzing the results of chromosomal alignment and of acting as an inter-cycle comparator. Transcription Factor Analysis using SELEX with High-Throughput sequencing (TFAST) was written in Java, and once sequences have been aligned to the genome of the target organism (for example, with BLASTn [14] or BOWTIE [15]), TFAST completes analysis in minutes on a personal computer. TFAST was designed to be useful to a broad range of biologists, so it employs an uncomplicated graphical interface to streamline its use. It is easy to install, requiring no special expertise in bioinformatics.

## Methods

### Peak identification

afSELEX-seq is performed using sheared genomic fragments, and the products of each of multiple cycles are subjected to high-throughput sequencing. Sequence data are aligned to the target chromosome using either BLASTn or any alignment method capable of producing output in SAM format (**Fig. 1b**). TFAST processes the same number of aligned reads from each cycle, thus reducing the impact of variability in sample read quality and completeness, to generate single column tables representing frequencies of aligned tags at every position within the target genome. TFAST then uses a modified sliding window algorithm (see [16] for a review of sliding window algorithms) to identify peaks in the final round of afSELEX-seq (**Fig. 1c**). The sliding window width is set to twice the estimated fragment length of an average library member and proceeds along the chromosome in single-nucleotide increments to identify strict maxima. To be considered peaks, maxima must be greater than two standard deviations above the mean frequency of the control (unselected) cycle within a range around the peak (distance set by user). Two standard deviations outside of the mean of the control (unselected) cycle frequencies represents the 95% confidence interval, so that the user may have confidence that peaks picked in this way are significant (p<0.05). Arguably, peak finding should proceed via one-tailed statistics, as the user is scoring enrichment specifically and not merely difference from a mean. Thus, two standard deviations may provide significance of p<0.025. Minimum spacing to identify individual peaks is set at a single fragment length, with the exceptions that deep valleys (<50% adjacent peak value) between peaks or adjacent peaks within 75% frequency of one another are both delimited as separate.

### Peak scoring

Frequencies at each peak position are compared across all cycles of afSELEX-seq, allowing the program to score peaks based on rate of enrichment as a proxy for binding affinity consistent with existing models of enrichment during SELEX (**Fig. 1c and d**) [3], [4], [7], [17], [18]. Sequences are given a cumulative score for each round in which they are able to enrich, so that sequences that remain strongly competitive for binding throughout afSELEX-seq achieve the highest scores. To reflect the increasing stringency of competition for binding in later rounds, the user should set scores for enrichment between cycles to be directly proportional to the bulk affinity of the library within each cycle. This causes TFAST to weigh later-round (*i.e.*, more stringent) enrichment more heavily than early-round enrichment, improving the discrimination of strongly-binding sequences.

TFAST then generates information on the relative representation of each peak, annotated as a raw final frequency and fraction of the area under a given peak in comparison to the total area under all other peaks in that cycle. TFAST also prints the genomic sequence under each peak (range determined by user) and outputs all data in tab-delimited format for downstream analysis. This format is designed to dovetail smoothly with methods for the discovery of DNA binding-site motifs. TFAST also automatically produces and analyzes sets of random sequence positions denoted “spoof” peaks, which can be used to improve motif discovery through counter-selection and background modeling. TFAST does not generate negative peaks, which are sometimes used to refine motif discovery, because counter-selection in afSELEX-seq produces a background of zero frequency between peaks. This means that negative peaks are artifacts of variation in control cycle sequencing and not indicative of especially poor regions of protein:DNA interaction.

### Binding site sequence length prediction

TFAST is designed to estimate the length of a putative region of protein:DNA interaction. Members of a dsDNA library should only have specific affinity for the protein of interest if they include the binding sequence. The full width of any peak over a single binding site will be twice the length of an average library member less the length of the binding site. TFAST calculates the full width of a peak as the range along the chromosome that includes 99% of the area under the peak, and then uses the width to calculate a predicted length for any binding site. Predicted length is included with the output of peak features.

### Validation and test data set

It was necessary to test the efficacy of TFAST against additional data sets. However, our dataset is the only extant set of its kind. ChIP-seq datasets produce short chromosomal reads, and SELEX-seq produces short random reads across cycles of enrichment. Our dataset, however, is the only one to produce short chromosomal reads across cycles of enrichment. Therefore, it was necessary to simulate a data set to validate TFAST. To simulate data, we parameterized our dataset and used the discovered trends to generate synthetic data. We first scrutinized the relationship between affinity for a binding site and the frequency above that site by estimating affinity of any given sequence within the genome using a simple linear algorithm that estimates similarity between a given sequence and a positional weight matrix:


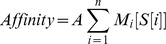


where *n* is the number of base pairs in the motif, *A* is a scaling factor, M*_i_*[c] gives the probability given by the motif of base pair c at position *i* in the motif, and S[*i*] gives the base pair in the sequence subject to analysis at position *i*.

We used the positional weight matrix generated in prior work (the parent motif) [6] and estimated similarity at every given site within the *E. coli* CFT073 genome as a function of frequency. All sites were ranked in order of descending similarity to the parent motif and binned into groups of 100. The average frequency at each site within each group of 100 was plotted against estimated affinity based on sequence similarity to the parent motif. Regression analysis was performed to generate an exponential model of the relationship between frequency and similarity. The scaling factor *A* was derived from observations of maximum and minimum affinity for species within the published afSELEX-seq data set estimated by this method, and set so that simulated sequences would enrich at similar cutoffs as that observed within the afSELEX-seq dataset (a value approximating 50% of maximum sequence identity). Additionally, for each of 100 best-quality peaks picked at random, the maximum frequency, mean frequency, and standard deviation was calculated and a Gaussian (normal) curve was generated from those values. Goodness of fit of each parent peak was compared to its derivative Gaussian distribution by least-squares regression analysis. An r^2^ was reported for each peak as it compared to its corresponding Gaussian derivative as an estimate for the normality of the distribution of peaks within our afSELEX-seq data set, to establish the distribution characteristics of our peaks for use in simulating peaks in our synthetic data sets.

Test data sets were generated from these values. For each test data set, a 5×10^6^ random bp genome is generated and a random 15–30 bp region of the synthetic genome is selected as the motif of interest. All sites are graded within the genome for their similarity to the randomly generated motif. The trend of similarity to parent motif to the frequency at that position derived from our afSELEX-seq dataset is then used to generate frequencies at each location within the chromosome, and Gaussian peaks are generated at each site. Total frequency within each test data set is normalized to 20×10^6^ reads to approximate the fixed number of reads in our afSELEX-seq data set per cycle sequenced.

100 test datasets were generated and TFAST was used to process each. The results of TFAST were compared to the actual motif that had been generated within each set by analyzing the highest peak in TFAST as well as the positional weight matrix generated from the most strongly enriching 100 best-quality peaks and motif discovery is performed by MEME [19].

## Results and Discussion

### TFAST accurately discriminates peaks in afSELEX-seq data

**Figure 2:**
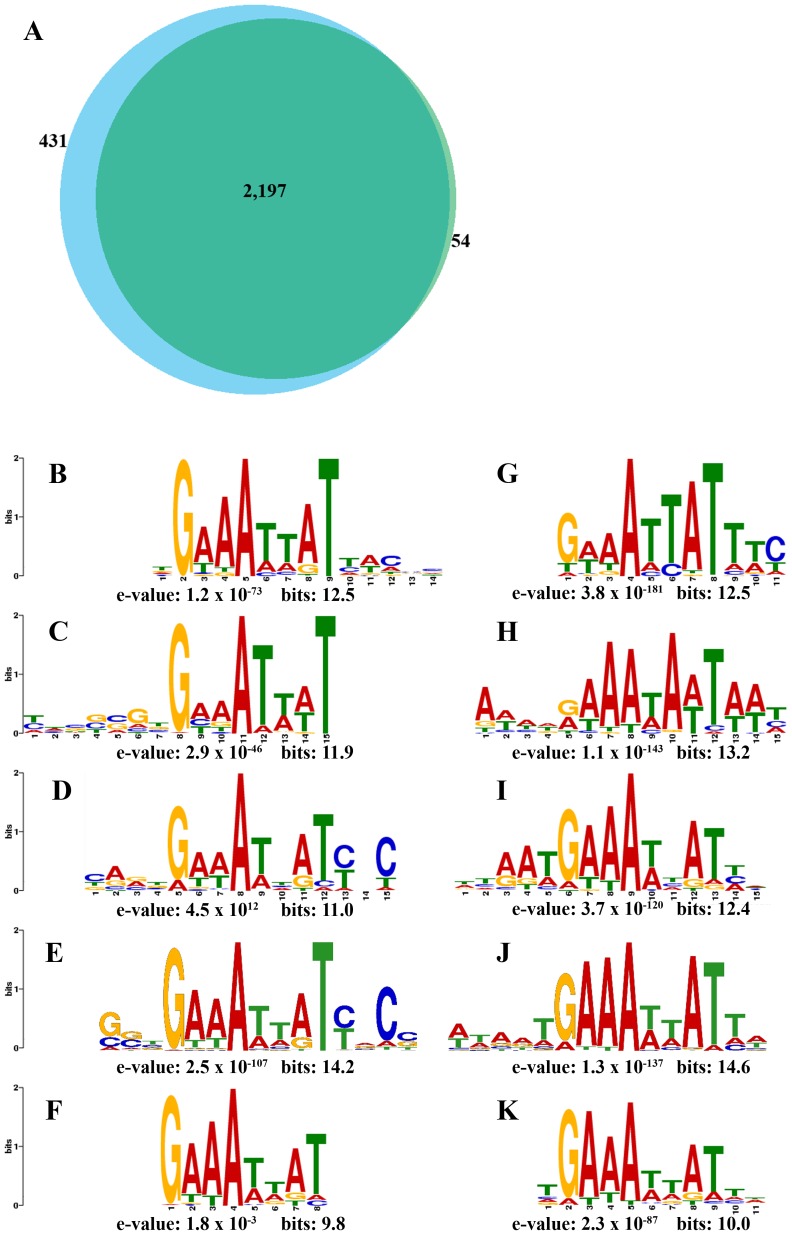
TFAST identifies peaks with discoverable motifs from afSELEX-seq data. TFAST and MACS were used to pick and evaluate peaks from our data set. (**A**) TFAST picked a total of 2,628 peaks, of which 2,197 covered 96% of the peaks identified by MACS in the final cycle of afSELEX-seq. Positional weight matrices generated in MEME instructed to search for a 15 bp motif using 200 sequences from (**B**) the 457 “Best” weight (most enriched) peaks, (**C**) the 888 next-best weight (second most enriched) peaks, (**D**) the 1,283 worst weight (least enriched) peaks, (**E**) all peaks called by TFAST pooled together and (**F**) 200 peaks called by MACS with the lowest false discovery rate (FDR). Sets of peaks from (**B–F**) were subjected again to analysis by MEME under the similar conditions but with the inclusion of a zero-order background Markov model to generate (**G–K**). E-value (the chance that a motif arose from a dataset by chance) and bit score (the total information content of a positional weight matrix) are shown below each logo.

To validate our peak calling method, we compared peak detection between TFAST and MACS [20] in an afSELEX-seq dataset generated in prior work [6]. We elected to compare to MACS because it is an established and well-vetted peak finding program designed for use with aligned high-throughput sequence data. For a recent discussion of MACS, see [21] and references within. TFAST called 96% of regions identified by MACS as peaks, and subdivided large peaks into multiple smaller ones (**Fig. 2a**). In detection of enriched regions, TFAST and MACS had a simple agreement of 0.98 and a κ value of 0.89, values generally considered to indicate near perfect agreement. The simple agreement of 0.98 and the κ of 0.89 between MACS and TFAST were calculated under the simplifying assumption that the 5,231,428 bp genome of *E. coli* strain CFT073 is divided into 26,157 candidate peak regions of 200 bp. Analysis assuming 13,079 candidate peak regions of 400 bp yields a simple agreement of 0.96 and a κ value of 0.88.

### TFAST peak grading correlates with informational density of discovered motifs

When TFAST was used to process our dataset, 2,628 total peaks were identified, of which 457 achieved the highest weight (“Best” peaks), or 17.4% of the total peaks called. When peaks are called without using the last or last two enrichment cycles, 4,255 and 7,274 peaks are identified, respectively, with a concurrent loss in specificity in identifying the “Best” peaks (31% and 100%, respectively) (**Table 1**). This demonstrates TFAST's unique advantage of using iterative cycles over simply the final set of peaks. Additionally, we instructed MEME [19] to discover 15 bp positional weight matrices (motifs) from peaks picked by TFAST or MACS (**Fig. 2b–k**). Peaks with the best, second best and worst weight scores identified by TFAST had e-values (an estimate for likelihood of a motif arising by chance) of 1.2×10^−73^, 2.9×10^−46^ and 4.5×10^12^, respectively, and informational content of 12.5, 11.9 and 11.0 bits, respectively (**Fig. 2b–e**). This indicates that TFAST was successful at stratifying classes of peaks over consistent sequences in the genome, and that positional weight matrix reliability was greatest in the “Best” peaks.

**Table 1:** Incorporation of increased SELEX cycle number improves peak discrimination.

Number of cycles used^a^	Total peaks called^b^	Number of “Best” peaks^c^	Percent “Best” peaks of total
3	2,628	457	17.4
2	4,255	1,350	31.7
1	7,274	7,274	100

a “Number of cycles used” does not include control.

b “Total peaks called” refers to peaks called using TFAST's algorithm.

c “Best” peaks are peaks with the highest weight scores for that set, as calculated by TFAST.

All peaks discovered by TFAST pooled together generated a positional weight matrix with an e-value of 2.5×10^−107^, an intermediate value that was less significant than “Best” peaks selected by TFAST (**Fig. 2e**). Furthermore, pooled peaks picked by MACS produced a logo with an e-value of 0.54, which was not significant. Using the 200 MACS peaks with the lowest false discovery rate (FDR), MEME was able to generate a positional weight matrix with an e-value of 1.8×10^−3^ and informational content of 9.8 bits (**Fig. 2f**). No other subset of MACS peaks (*i.e.*, peaks with lowest p-value, greatest fold enrichment, etc) generated a logo with an e-value less than 1. This is a substantially larger e-value than that generated from the peaks discovered in TFAST, likely because the average width of peaks picked by MACS was 503±a standard error of 9.71, whereas all peaks picked by TFAST were under 200 bp wide. These findings support the use of the method employed by TFAST of picking many narrow peaks based on absolute local maxima, coupled with fragment length, rather than statistical modeling of aligned sequences.

### Background models derived from TFAST improve motif discovery

To validate the use of spoof peaks (described in **methods**) to improve motif discovery, we generated a zero-order Markov model from the spoof output of TFAST. When we incorporated the model into our motif discovery using MEME, the information content of the motifs rose by an average of 0.78 bits or 6.3%, and e-value fell by an average of 8.2×10^−133^ for peaks picked by TFAST (**Fig. 2g–j**). Additionally, the Markov-corrected 15 bp motif from the “Best” weight sequences picked by TFAST most accurately predicted the 15 bp core of the previously validated binding site GTTATTTTAAC [6]. Use of the Markov model also strengthened the motif generated from MACS, improving information content from 9.8 to 10.0 bits or 2.0% and the e-value from 1.8×10^−3^ to 2.3×10^−87^ (**Fig. 2f, 2k**). Overall, this supports the notion that using an accurate background model generated from TFAST can improve the ability to discover significant motifs in downstream peak analysis, and improves total motif informational content.

### TFAST can accurately predict binding site sequence length

In our data set, TFAST predicted a binding site width for the “Best” peaks of 20.88 bp ± a standard deviation of 10.33 bp, consistent with the motifs discovered. Additionally, TFAST predicted the binding site for the peak in the *flhD* promoter of CFT073 for PapX to be 28 bp. Experimentally, PapX binds a 29 bp fragment of the *flhD* promoter, but not a truncated 21 bp fragment [6] **.** The consistency between the prediction made by TFAST and the experimentally verified binding site indicates that TFAST is capable of predicting binding site length from afSELEX-seq data.

### TFAST reliably discovers motifs in synthetic datasets

To generate our simulated afSELEX-seq datasets, we sought to determine trends in peak distribution and shape to accurately simulate real afSELEX-seq data. To determine shape, we analyzed 100 random best-quality peaks from the fourth cycle of enrichment from our afSELEX-seq dataset generated in previous work [6] for Gaussian fit by a least-squares regression analysis. 52% of peaks had an r^2^ value of 0.99 or better, and the average r^2^ of all the peaks was 0.92 with a standard deviation of 0.18. These results support the use of Gaussian distributions for the peaks generated in our synthetic dataset.

**Figure 3:**
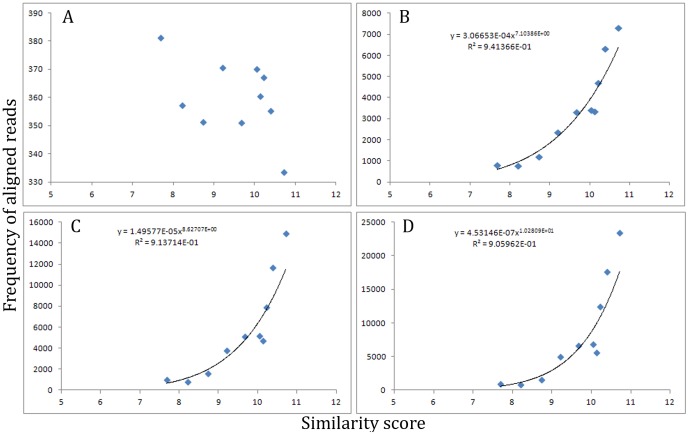
Similarity score of sequences to a motif is predictive of frequency. Similarity of each chromosomal position to the discovered (parent) motif was calculated as a stand in for relative affinity using a linear nucleotide position matching algorithm (see **methods**). All positions were ranked and the mean similarity score of groups of 100 with serially lower affinity scores were plotted against the mean frequency of those positions. The x-axis left margin is 5 because a bit score of 0 (the statistical similarity between random sequences) should be 5.25 for the parent motif under investigation. The rightmost edge of the x-axis represents the discovered highest similarity (11.25) of a single region of the chromosome to the discovered parent motif, representing a best fit and thus maximal similarity of the set. (**a**) The similarity scores in the initial (unselected) library did not produce a statistically significant trend when subjected to regression analysis. (**b–d**) The similarity scores from sequential sequence cycles of afSELEX-seq against their corresponding frequencies revealed exponential regression models (black lines) with r^2^ values all over 0.90.

We reasoned that the similarity of sites to the true binding motif would directly correlate with affinity, so that sites with relatively high similarity to the true binding site would have relatively high affinity, and sites with relatively low similarity would have relatively low affinity. It follows that sites with relatively high affinity would undergo selection during afSELEX-seq and thus enrich in frequency, and sites with relatively lower affinity would not enrich as well and thus their frequency would be less well represented. To test our hypothesis, we computed similarity scores for each region of the *E. coli* CFT073 genome to the binding site motif discovered for PapX in previous work [6] for each cycle of afSELEX-seq sequenced using a linear matching algorithm (see **methods**). Each region was ranked for similarity, and the average similarity and frequency for groups of 100 regions with sequentially lower similarity were compared for each sequenced cycle of afSELEX-seq (**fig. 3a–d**). In the control (unselected) library, there was no correlation between predicted sequence affinity for PapX and frequency, consistent with the fact that no enrichment based on affinity had yet occurred (**fig. 3a**). In each of the enriched cycles, an exponential function described the correlation between predicted affinity of a region and the observed frequency of enrichment over that site (**fig. 3b–d**). The r^2^ values of the affinity-to-frequency functions indicate that over 90% of the frequency (and thus enrichment) of a sequence is explainable by its similarity to a binding site. The similarity-frequency regressions increase in steepness between rounds, indicating that sequences with relatively more similarity to the discovered binding motif consistently display relatively higher affinity across rounds. Together, these data indicate that sequence similarity to a binding motif is a reliable predictor for frequency within a cycle afSELEX-seq, and thus were dependable models for our synthetic data sets. In addition, the number of peaks generated by this method per data set was always within the same order of magnitude as those discovered in our afSELEX-seq data, further supporting the use of these parameters to simulate afSELEX-seq data.

We used the parameters discovered from our experimentally derived afSELEX-seq data set to generate 100 synthetic datasets. Within each data set, each region of the chromosome was assigned a frequency that corresponded to that region's similarity to the binding motif picked randomly for the given simulated data set, using the affinity algorithms derived from our afSELEX-seq data in conjunction with the similarity algorithm (described in **methods**). TFAST was then used to process each simulated data set and the results were analyzed for accuracy of predicted binding site length and sequence content of predicted binding site motif.

Across all 100 test data sets, 75% the length of the binding site was within 2 standard deviations of the average predicted length derived by TFAST from the 100 most enriched best-quality peaks from each respective set. 86.7% of binding site length predictions were within 1 standard deviation of that predicted by TFAST for motifs 25 bp and shorter. In parallel we analyzed the sequence motifs derived from the test dataset. Sequences from the 100 most enriched best-quality peaks detected by TFAST within each dataset produced motifs when processed by MEME [19] that were compared to the motif randomly generated for that specific dataset. Motifs generated by MEME from sequences selected by TFAST were 94.7% identical to the motifs generated by in the simulated data set where they aligned. On average, motifs generated from TFAST covered 87.3% of the length of motifs overall, and covered 100% of the length of motifs 20 bp or shorter. Sequence content alone was insufficient to cover entire binding sites, underscoring the need to pair binding site length predictions with sequence content predictions to accurately predict binding sites. We concluded that TFAST is able to reliably discover and predict binding site motifs out of large afSELEX-seq –like data sets, and that it is well-suited as a tool for analyzing this type of data.

### Conclusions

Here we present TFAST, an easy-to-use tool for rapidly and accurately analyzing data generated by afSELEX-seq to discover and characterize transcription factor binding sites. Currently, no other software is designed to analyze afSELEX-seq. The peak finding component of TFAST compares favorably to that of MACS. For use with afSELEX-seq, TFAST outperforms MACS in generating significant motifs. The scheme TFAST employs for grading and analyzing peaks uniquely leverages the cyclical nature of SELEX and direct protein:genomic dsDNA interactions to accurately and sensitively predict binding site sequence and length, as demonstrated by the consistent concurrence between predictions generated by TFAST and our experimental findings [6].

afSELEX-seq offers an alternative to current methods of discovering DNA binding sites, and requires no antibody generation (unlike ChIP-seq (reviewed in [22] and references therein)) or complex biological screens (unlike bacterial 1-hybrid systems [23]). Large libraries of purified, tagged bacterial proteins of unknown function currently exist at institutions participating in the Protein Structure Initiative [24]. This is sufficient material to run afSELEX-seq, and with only minor modifications the binding sites of hundreds if not thousands of transcription factors could be quickly elucidated.

### Availability

TFAST is implemented in Java and supported on MS Windows and Mac OSX. TFAST was designed in compliance with the GNU GPL. TFAST (**file S1**), source code (**file S2**), documentation with instructions for use (**files S3 and S4**), and example output (**fig. S1**) are freely available for download at http://www-personal.umich.edu/~hmobley/ or http://sourceforge.net/projects/tfast/files/ and are included in the supplementary information.

## Supporting Information

Figure S1 **Example output.** The output of TFAST is generated in a tab-delimited format. Displayed is how a typical output of TFAST analysis ought to appear.
(JPG)

File S1 **Executable files of TFAST.** The executable files needed to run TFAST, compressed in .zip format.
(ZIP)

File S2 **Source files of TFAST.** The source files for TFAST, compressed in .zip format.
(ZIP)

File S3 **TFAST instructions for use.** Documentation and instructions for implementation and use of TFAST, in .txt format.
(TXT)

File S4 **TFAST instructions for use.** Documentation and instructions for implementation and use of TFAST, in .rtf format.
(RTF)
