# Supplementary figures and images for: A Novel Approach for Transcription Factor Analysis Using SELEX with High-Throughput Sequencing (TFAST)
Source: PLoS One. 2012 Aug 3;7(8):e42761. doi: 10.1371/journal.pone.0042761 (PMC3430675; doi:10.1371/journal.pone.0042761)

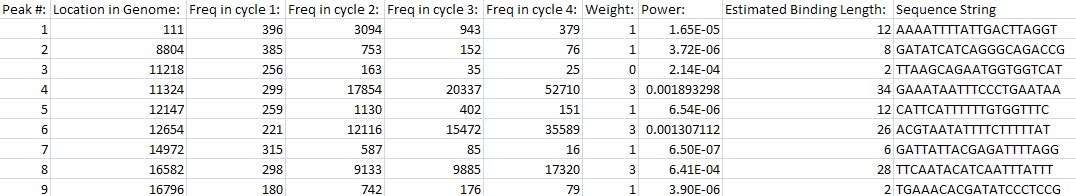

Supplement: Figure S1 — Example output. The output of TFAST is generated in a tab-delimited format. Displayed is how a typical output of TFAST analysis ought to appear. (JPG) [file pone.0042761.s001.jpg]

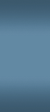

Supplement: File S2 — Source files of TFAST. The source files for TFAST, compressed in .zip format. (ZIP) [file pone.0042761.s003.zip › Source/File Type Conversion/doc/resources/background.gif]

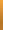

Supplement: File S2 — Source files of TFAST. The source files for TFAST, compressed in .zip format. (ZIP) [file pone.0042761.s003.zip › Source/File Type Conversion/doc/resources/tab.gif]

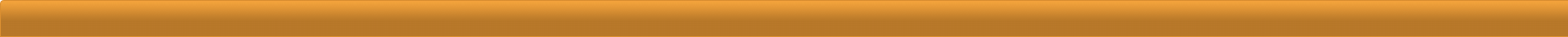

Supplement: File S2 — Source files of TFAST. The source files for TFAST, compressed in .zip format. (ZIP) [file pone.0042761.s003.zip › Source/File Type Conversion/doc/resources/titlebar.gif]

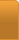

Supplement: File S2 — Source files of TFAST. The source files for TFAST, compressed in .zip format. (ZIP) [file pone.0042761.s003.zip › Source/File Type Conversion/doc/resources/titlebar_end.gif]
