# Supplementary material for: A Novel Approach for Transcription Factor Analysis Using SELEX with High-Throughput Sequencing (TFAST)
Source: PLoS One. 2012 Aug 3;7(8):e42761. doi: 10.1371/journal.pone.0042761 (PMC3430675; doi:10.1371/journal.pone.0042761)
Supplement: File S1 — Executable files of TFAST. The executable files needed to run TFAST, compressed in .zip format. (ZIP) [file pone.0042761.s002.zip › TFAST/TFAST ReadMe RTF.rtf]

TFAST ReadMe
      
Overview of TFAST

TFAST was originally adapted from a series of algorithms used to analyze SELEX with high-throughput sequencing data. For a review of the aptamer-free SELEX-seq protocol used, see Reiss, D.J and Mobley, HLT. "Determination of the target sequence bound by PapX, a repressor of bacterial motility, in the flhd promoter using SELEX and high-throughput sequencing (DOI 10.1074/jbc.M111.290684). In order to make optimal use of TFAST, first the initial (unselected) library used for SELEX must be sequenced, as well as selected subsequent cycles. Once sequence data has been procured, they can be processed using TFAST. Alternatively, the user may process files as she or he wishes and enter completed files into the TFAST workflow, so long as files are properly formatted. Included here are instructions for the use of TFAST and the file format used at each stage, so that the user can integrate features as they see fit.
Dependencies: TFAST is written in Java. Java must be installed to execute the .jar files.

Terms
“Probe length” or “fragment length” refer to the average size of fragment submitted for sequencing in each round of SELEX sequenced.
“Upper limit of reads” refers to the number of reads aligned in the cycle of SELEX in which the greatest number of reads was aligned to the target sequence.
“N” and “#” are placeholders for integer values.
“Power” refers to the area under peaks and is a surrogate for representation of a peak in the final cycle of SELEX sequenced
“Weight” is an indicator of how well a given peak enriched. Weights per cycle of SELEX are determined by the user, and should reflect the bulk binding characteristics of the library (ie, should increase with stringency as cycles are iteratively enriched).


Quick use guide
1. Run fileconversion.jar on Illumina _fastq.txt formatted sequence folder
2. Run folder blast.jar on the resulting /fasta directory from step “1”
3. Run freq counter.jar on the resulting /blast directory from step “2”
3. Alternatively, run sam freq counter.jar on .SAM files generated by the user using another aligner.
4. Run peak finder.jar on the resulting freq_n.txt files generated in either step “3”
5. Run evaluation.jar on the resulting peak.txt or spoof.txt files generated in step “4”


Instructions for use
The first step in analyzing aptamer-free SELEX-seq data is to align the reads generated by next-generation sequencing (NGS) to a target sequence (eg, a chromosome). TFAST can accept alignment in the form of BLASTn output or .SAM files. BLASTn tends to generate a very complete alignment of NGS reads to a target sequence, but tends to be computationally heavy and may take some time. To align sequences using BLASTn follow the steps as outlined. TFAST can also accept .SAM alignment files, which can be generated very quickly using programs such as BOWTIE. To use .SAM files, enter the workflow at step “alternate step 3 sam freq counter”.

Step 1   fileconversion.jar
BLASTn requires that sequences are in FASTA format, so the first program in the TFAST workflow, fileconversion.jar, converts Illumina high-throughput sequence data (typically in a form similar to “s_#_1_0#_qseq.txt”, ie “s_5_1_0001_qseq.txt” or “s_1_1_0120_qseq.txt”) into FASTA format. Fileconversion currently only supports Illumina's _qseq.txt format. Fileconversion.jar only coverts uninterrupted reads from _qseq.txt files into FASTA format; partial, degenerate or incomplete reads will not be converted. Fileconversion.jar does not take read quality (PHRED score) into account when converting sequences. For files in fastq format, existing open-source resources exist for converting sequences to FASTA (such as the Galaxy suite of tools, http://usegalaxy.org)
To use fileconversion.jar, execute the application. This will open the command window and prompt the user to enter the directory that contains _qseq.txt files.
Input: Directory of _qseq.txt files, each line of the format: 	
HWUSI-EAS1707	1	6	1	1871	1088	0	1AAACAATTGACAATGATTATCATTTGCATTAAAAAGACTACGTAGTATTTTTATTTCATGAGGAACTATACCCGCCAGCA	fffaWd`ff]ffd^_SaYa^fffdfcdRccacc\^[]`dccW^ccRb^bbccccccccfccacc_cc[WWdbb````BBB
1
Output: /fasta subdirectory of the original directory, with a file corresponding to each _qseq.txt file (naming in line with fasta_ s_#_1_000#_qseq.txt), with lines from the _qseq.txt files formatted as follows (corresponding to the above input line):     
>18711088
AAACAATTGACAATGATTATCATTTGCATTAAAAAGACTACGTAGTATTTTTATTTCATGAGGAACTATACCCGCCAGCA
Notes: If the raw sequence was not determinable at one point (indicated by one or more '.' characters instead of A/C/G/T), the read will be discarded.

Step 2   Folder blast.jar
Requirements: Download and install the appropriate version of BLASTn for your operating system. BLASTn is a utility provided by the NCBI at no cost at (http://www.ncbi.nlm.nih.gov/books/NBK1762).
Folder blast.jar uses FASTA formatted sequences, provided by the user or generated by fileconversion.jar in Step 1. The user will also have to provide a sequence file of the genome of the organism being investigated, in .fna format. .fna files can be found on the NCBI website (http://www.ncbi.nlm.nih.gov/pubmed/).
Folder blast.jar uses a shell script to run BLASTn on the FASTA formatted sequences against the appropriate .fna file of interest. To use folder blast.jar, execute the application. This will open the command window and prompt the user to enter the directory containing the FASTA formatted sequence files as well as the .fna genome sequence file. The result of folder blast.jar are BLAST-formatted .txt files (naming in line with blast_fasta_ s_#_1_000#_qseq.txt). Check the NCBI website for tips on streamlining very large BLAST sets (http://www.ncbi.nlm.nih.gov/books/NBK1762).

Input: Directory of FASTA formatted sequences, as described above. Each .txt file in the directory will be run with BLAST - be sure to include only FASTA sequence files.
	
Output: /blast subdirectory of the /fasta, with a file corresponding to each FASTA_ s_#_1_000#_qseq.txt file. Output is a series of BLAST files corresponding to each FASTA sequence read. Also requires a plain sequence .fna file.
      
Step 3    freq counter.jar
Freq counter.jar uses results of local BLAST files to generate a position-frequency table, provided by the user or by running folder blast.jar. Frequency tables can be generated by the user using other utilities or program packages (see description of _freq.txt format, below). Freq counter.jar takes the aligned sequences from folder blast.jar and counts the number of sequence reads that overlap at each nucleotide within the chromosome of interest. This results in a frequency table (displayed freq_#.txt), which is a tab-delimited single column table the length of the target sequence (sequence aligned to, ie chromosome or genome) that indicates frequencies at each position.
To use freq counter.jar, execute the application. This will open the command window and prompt the user to enter the directory containing the files produced in the local BLAST of sequences, the genome's .fna file, the estimated upper bound on the number of aligned reads and the cycle number. Cycle number is “1” for the control cycle (the unselected cycle, i.e. the starting library of sequences), “2” for the second cycle to be analyzed, etc. The estimated upper bound for the number of aligned reads allows freq counter.jar to normalize the number of reads processed. This ensures that freq counter.jar will not bias frequency files due to variation in raw number of reads between or within lanes during sequencing. Blast sample counter.jar can estimate the number of aligned reads for BLASTn aligned files. To use it, execute the program, select the directory containing the blast_ files and run. The fields will populate automatically.

Input: Directory of BLAST results, as described above. Each .txt file in the directory will be included in the frequency table generation so be sure to include only relevant BLAST results. Also requires a plain sequence .fna file.

Output: /freq subdirectory of the /blast directory, with a freq_n.txt file which is a list of integers, with the nth integer corresponding to the frequency of the nth location in the genome.

Alternate step 3  sam freq counter.jar
A user may choose to use a different method of aligning their NGS data to a target sequence. The easiest way to incorporate alternative (non-BLASTn) alignments into the TFAST workflow is to generate .SAM files (such as by using BOWTIE). There are several good, freely available NGS alignment tools. Many tools of this type require FASTQ formatted sequences (instead of Illumina's _qseq.txt format). An easy way to convert _qseq to FASTQ is to use Bash (the command line in Cygwin and Unix-based systems, ie Linux, OSX, etc), navigate to the directory containing the _qseq.txt files and enter the command (after the $):
for ((x=1;x<=8;x+=1)); do cat s_"$x"_1_*_qseq.txt | awk -F '\t' '{gsub(/\./,"N", $9); if ($11 > 0) printf("@%s_%04d:%s:%s:%s:%s#%s/%s\n%s\n+%s_%04d:%s:%s:%s:%s#%s/%s\n%s\n",$1,$2,$3,$4,$5,$6,$7,$8,$9,$1,$2,$3,$4,$5,$6,$7,$8,$10)}' > s_"$x"_sequence.fastq; done      
Resulting files should be .txt files in FASTQ format.
Once the user has generated .SAM files using their alignment tool of choice, execute sam freq counter.jar. This will open the command window and prompt the user to enter the directory containing .SAM files for a particular cycle, the genome .fna file, the upper bound on number of aligned reads (usually given in aligner reports), the cycle number, the fragment length subjected to sequencing, and the read length (number of bases sequenced/read per fragment). Sam freq counter.jar will then generate freq_n.txt files as described in step 3.

Step 4 – peak finder.jar
Peak finder.jar uses the frequency tables of the control library and the final round of SELEX (that is, the most enriched round that was sequenced). Frequency tables can be generated using TFAST or generated by the user. Peak finder.jar determines local maxima, and rejects peaks that are less than two standard deviations above the mean of an n-bp window (set by the user) around that peak position in the control library. Peak finder.jar also rejects peaks that were two standard deviations above the mean in the control library, to reduce bias from aberrant variation in the control cycle being counted as peaks. Peak finder.jar examines the sequence within a fragment length (probe length) of each peak to determine whether or not a given peak is an aberration or represents a true maxima for that region.
To use peak finder.jar, execute the application. This will open the command window and prompt the user to enter the frequency file of the most enriched SELEX cycle, the frequency file of the control library, the .fna file of the genome of interest, a manually set threshold for peaks in the final cycle (optional), the threshold window (we suggest a value equal to or greater than probe length) and the estimated fragment length (probe length). Probe length can be determined empirically by running the library on a high-resolution gel, or when the initial library is selected for SELEX analysis.
Peak finder.jar produces two files – Peak.txt and Spoof.txt. Peak.txt is a .txt file containing a single column of chromosomal positions corresponding to predicted peaks. The user may elect to use his/her own peak finding algorithms, in which case, format the output similarly to proceed to Step 5. Spoof.txt is a similarly formatted file that contains chromosomal positions generated randomly. Spoof.txt can be used as a control file to validate results and improve downstream data analysis when using motif finding software and to generate background models of genome sequence behavior and nucleotide representation.
TFAST can also find minima instead of maxima if the user desires. In most SELEX experiments, this will result in a vast number of minima as SELEX tends to produce zero frequency between peaks. This option is useful for knockout experiments, such as transposon mutagenesis studies coupled with high-throughput sequencing. When selecting minima, TFAST subtracts the final cycle from the control cycle and counts regions that are two standard deviations of the control cycle (within the set window) above zero as peaks.

Input: freq_n.txt (where n is the number of cycles), freq_1.txt (the control library), a plain sequence .fna file, a set threshold if necessary, threshold window and the average probe (fragment) length.
      
Output: /peak subdirectory, with a peak.txt file and a spoof.txt file.

Step 5 – Evaluaton.jar
Evaluaton.jar uses peak positions determined in Step 4 or provided by the user to grade peaks. Before using evaluaton.jar, place all freq_#.txt files of interest (including the control) into a single directory. To use evaluator.jar, execute the application. This will open the command window and prompt the user to enter the directory containing all freq_#.txt files, the .fna file of the genome of interest, the peak.txt file, weights for every round, average probe length (fragment length) and the length of sequence around each peak to be included in the output.
Weights for every round should be delimited with commas. Every cycle of SELEX selects for the most strongly binding species in a pool. Thus, as cycles continue, the requirements for binding increase. Therefore, we advise the user to use a scheme that weighs enrichment in later rounds higher than enrichment in earlier rounds. For example, for a SELEX with three rounds of enrichment (control library, enriched1, enriched2 and enriched3), one scoring schema could be “1, 1.5, 2”. “1,1,1” will result in equal weights in every round, which does not reflect the increasing stringency of competitive binding in the DNA fragment libraries. Ideally, a scoring scheme for weights between rounds will be based on the fraction of input species that are recovered after each round in the SELEX experiment, reflecting the bulk affinity of the library for the protein of interest. These considerations are less of a concern if the user performs “fixed stringency SELEX”.
Once completed, evaluaton.jar will produce a tab-delimited table called “output.txt”, including data on each peak.
-The first (left-most) column describes the peak number, which for most users can be disregarded (useful for testing the program internally).
-The second column describes the location of the peak within the chromosome, based on the .fna file provided.
-The third through nth columns describe the frequency at that chromosomal position for each SELEX cycle amplified, where “n” is the cycle number (1 = the control library, 2 = first enrichment entered, etc).
-The (n+1)th column describes the weight of a peak. Weight is determined by adding the “weight” values when the frequency at a position increased between cycles, and by adding 0 when the frequency at a position remained unchanged or declined. High weights correspond to strongly enriching sequences.
-The (n+2)th column describes the power of a peak. Power represents the area under a peak as compared to the total area under peaks for the final cycle sequence. Power is another way to grade how strongly a fragment has enriched relative to every other fragment.
-the (n+3)th column describes an estimated binding site length. This is determined by finding the bounds under a peak that includes 99% of the peak area, and subtracting that distance from twice the probe length. Because probe length will vary slightly for technical reasons, it is best to use an average of several peaks to assess the theoretical binding length for a site. This can be useful for determining cases of multiple adjacent binding sites as well as for discarding large repeated regions.
-The (n+4)th column describes The chromosomal sequence that falls under a given peak, with the amount of sequence included determined by the user. The length included should conform to the standards of any downstream analysis planned.

Evaluation.jar can also analyze the quality of minima (valleys) instead of peaks. This utility is useful for knockout experiments that incorporate NGS, but is unlikely to yield results in SELEX data.
	
Input: A folder containing only the freq_1.txt through freq_n.txt files, with every file from 1 to n included. Also, a plain sequence .fna file, average probe length, length of flanking sequence to be printed, and a list of weights.      

Output: /output subdirectory, with a output.txt file as referenced above.


A dataset of SELEX data using high-throughput sequencing is provided in supplemental data. Feel free to make use of them as a practice set. The files are from an actual experiment that used the algorithms found in TFAST to determine and experimentally validate a novel, unique binding site for a transcription factor (PapX) in E. coli.
