# Supplementary material for: A Novel Approach for Transcription Factor Analysis Using SELEX with High-Throughput Sequencing (TFAST)
Source: PLoS One. 2012 Aug 3;7(8):e42761. doi: 10.1371/journal.pone.0042761 (PMC3430675; doi:10.1371/journal.pone.0042761)
Supplement: File S2 — Source files of TFAST. The source files for TFAST, compressed in .zip format. (ZIP) [file pone.0042761.s003.zip › Source/File Type Conversion/doc/class-use/OpenFileAction.html]

Uses of Class OpenFileAction


JavaScript is disabled on your browser.


- Package
- Class
- Use
- Tree
- Deprecated
- Index
- Help

- Prev
- Next

- Frames
- No Frames

- All Classes

## Uses of Class OpenFileAction

No usage of OpenFileAction

- Package
- Class
- Use
- Tree
- Deprecated
- Index
- Help

- Prev
- Next

- Frames
- No Frames

- All Classes
