# Supplementary material for: A Novel Approach for Transcription Factor Analysis Using SELEX with High-Throughput Sequencing (TFAST)
Source: PLoS One. 2012 Aug 3;7(8):e42761. doi: 10.1371/journal.pone.0042761 (PMC3430675; doi:10.1371/journal.pone.0042761)
Supplement: File S2 — Source files of TFAST. The source files for TFAST, compressed in .zip format. (ZIP) [file pone.0042761.s003.zip › Source/File Type Conversion/doc/main.html]

main


JavaScript is disabled on your browser.


- Package
- Class
- Use
- Tree
- Deprecated
- Index
- Help

- Prev Class
- Next Class

- Frames
- No Frames

- All Classes

- Summary:
- Nested |
- Field |
- Constr |
- Method

- Detail:
- Field |
- Constr |
- Method


## Class main

- java.lang.Object
- - main

- ---

    

  ```
  public class main
  extends java.lang.Object
  ```

- - ### Constructor Summary

    Constructors

    | Constructor and Description |
    | `main()` |
  - ### Method Summary

    Methods

    | Modifier and Type | Method and Description |
    | `static void` | `main(java.lang.String[] args)` |

    - ### Methods inherited from class java.lang.Object

      `equals, getClass, hashCode, notify, notifyAll, toString, wait, wait, wait`

- - ### Constructor Detail


    - #### main

      ```
      public main()
      ```
  - ### Method Detail


    - #### main

      ```
      public static void main(java.lang.String[] args)
      ```


- Package
- Class
- Use
- Tree
- Deprecated
- Index
- Help

- Prev Class
- Next Class

- Frames
- No Frames

- All Classes

- Summary:
- Nested |
- Field |
- Constr |
- Method

- Detail:
- Field |
- Constr |
- Method
