# Supplementary material for: A Novel Approach for Transcription Factor Analysis Using SELEX with High-Throughput Sequencing (TFAST)
Source: PLoS One. 2012 Aug 3;7(8):e42761. doi: 10.1371/journal.pone.0042761 (PMC3430675; doi:10.1371/journal.pone.0042761)
Supplement: File S2 — Source files of TFAST. The source files for TFAST, compressed in .zip format. (ZIP) [file pone.0042761.s003.zip › Source/File Type Conversion/doc/OpenFileAction.html]

OpenFileAction


JavaScript is disabled on your browser.


- Package
- Class
- Use
- Tree
- Deprecated
- Index
- Help

- Prev Class
- Next Class

- Frames
- No Frames

- All Classes

- Summary:
- Nested |
- Field |
- Constr |
- Method

- Detail:
- Field |
- Constr |
- Method


## Class OpenFileAction

- java.lang.Object
- - javax.swing.AbstractAction
  - - OpenFileAction

- All Implemented Interfaces:
  :   java.awt.event.ActionListener, java.io.Serializable, java.lang.Cloneable, java.util.EventListener, javax.swing.Action

  ---

    

  ```
  public class OpenFileAction
  extends javax.swing.AbstractAction
  ```

  See Also:
  :   Serialized Form

- - ### Field Summary

    - ### Fields inherited from interface javax.swing.Action

      `ACCELERATOR_KEY, ACTION_COMMAND_KEY, DEFAULT, DISPLAYED_MNEMONIC_INDEX_KEY, LARGE_ICON_KEY, LONG_DESCRIPTION, MNEMONIC_KEY, NAME, SELECTED_KEY, SHORT_DESCRIPTION, SMALL_ICON`
  - ### Method Summary

    Methods

    | Modifier and Type | Method and Description |
    | `void` | `actionPerformed(java.awt.event.ActionEvent evt)` |

    - ### Methods inherited from class javax.swing.AbstractAction

      `addPropertyChangeListener, getKeys, getPropertyChangeListeners, getValue, isEnabled, putValue, removePropertyChangeListener, setEnabled`
    - ### Methods inherited from class java.lang.Object

      `equals, getClass, hashCode, notify, notifyAll, toString, wait, wait, wait`

- - ### Method Detail


    - #### actionPerformed

      ```
      public void actionPerformed(java.awt.event.ActionEvent evt)
      ```


- Package
- Class
- Use
- Tree
- Deprecated
- Index
- Help

- Prev Class
- Next Class

- Frames
- No Frames

- All Classes

- Summary:
- Nested |
- Field |
- Constr |
- Method

- Detail:
- Field |
- Constr |
- Method
