# Supplementary material for: A Novel Approach for Transcription Factor Analysis Using SELEX with High-Throughput Sequencing (TFAST)
Source: PLoS One. 2012 Aug 3;7(8):e42761. doi: 10.1371/journal.pone.0042761 (PMC3430675; doi:10.1371/journal.pone.0042761)
Supplement: File S2 — Source files of TFAST. The source files for TFAST, compressed in .zip format. (ZIP) [file pone.0042761.s003.zip › Source/File Type Conversion/doc/serialized-form.html]

Serialized Form


JavaScript is disabled on your browser.


- Package
- Class
- Use
- Tree
- Deprecated
- Index
- Help

- Prev
- Next

- Frames
- No Frames

- All Classes

# Serialized Form

- ## Package &lt;Unnamed&gt;

  - ### Class gui extends javax.swing.JFrame implements Serializable

    - ### Serialized Fields

      - #### contentPanel

        ```
        javax.swing.JPanel contentPanel
        ```
      - #### directoryTextField

        ```
        javax.swing.JTextField directoryTextField
        ```
  - ### Class OpenFileAction extends javax.swing.AbstractAction implements Serializable

    - ### Serialized Fields

      - #### frame

        ```
        javax.swing.JFrame frame
        ```
      - #### chooser

        ```
        javax.swing.JFileChooser chooser
        ```
      - #### update

        ```
        javax.swing.JTextField update
        ```

- Package
- Class
- Use
- Tree
- Deprecated
- Index
- Help

- Prev
- Next

- Frames
- No Frames

- All Classes
